# Two-Stage Transvenous Implantable Cardioverter-Defibrillator Lead Extraction Guided by 3D Transesophageal Echocardiography in Severe Cardiomyopathy: A Case Report

**DOI:** 10.7759/cureus.113030

**Published:** 2026-07-20

**Authors:** Tejash Sikka, Tarick Ahmad, Sridhar Musuku

**Affiliations:** 1 Anesthesiology, Albany Medical College, Albany, USA

**Keywords:** anesthesia management, cardiac device infection, cardiac implantable electronic device, implantable cardioverter defibrillator, lead extraction, mrsa bacteremia, nonischemic cardiomyopathy, transesophageal echocardiography, transvenous lead extraction, two stage lead extraction

## Abstract

Transvenous pacer and defibrillator lead extraction is the standard treatment for cardiac implantable electronic device (CIED) infection but carries significant procedural risk, particularly in patients with advanced cardiomyopathy, active infection, and long-standing lead dwell times.

A 54-year-old male with nonischemic cardiomyopathy (ejection fraction <20%) and end-stage renal disease on hemodialysis presented with methicillin-resistant *Staphylococcus aureus* (MRSA) bacteremia in the setting of a biventricular implantable cardioverter-defibrillator (ICD) placed 13 years earlier. Echocardiography demonstrated severe systolic dysfunction and a mobile echodensity consistent with vegetation on a pacemaker lead near the superior vena cava-right atrial junction, necessitating complete device extraction. Initial laser-assisted extraction in the operating room under general anesthesia with invasive monitoring and transesophageal echocardiography (TEE) guidance successfully removed one lead, but a second lead could not be fully extracted despite attempted removal. The patient was maintained on targeted intravenous antibiotics, and completion extraction was performed two weeks later; however, multiple conventional snaring attempts were unsuccessful due to lead orientation and adherence within the right atrium. Three-dimensional TEE (3D-TEE) was subsequently used to improve spatial visualization, enabling successful femoral snare capture and removal of the retained lead without complication.

This case highlights the importance of procedural planning and advanced imaging in high-risk lead extraction, as a staged approach may mitigate risk while 3D-TEE provides critical real-time spatial guidance when conventional techniques fail.

## Introduction

Cardiac implantable electronic device (CIED) infection is an accepted indication for complete device and lead removal, with delayed extraction associated with adverse outcomes [1]. Current guidelines from the Heart Rhythm Society and American Heart Association recommend complete percutaneous extraction of all hardware, including leads and the generator, in patients with CIED infection confirmed by bloodstream infection or device/lead vegetation, given the risk of persistent bacteremia, relapsing infection, and endocarditis with device retention [1,2]. Transvenous extraction remains the recommended approach despite carrying significant procedural risks, particularly in patients with severe cardiomyopathy, active infection, or prolonged device and lead dwell times. Over time, chronic leads develop dense fibrotic adhesions to surrounding vascular structures, substantially increasing the risk of complications during extraction, including myocardial perforation, vascular injury, and hemodynamic instability [2].

The role of transesophageal echocardiography (TEE) in monitoring intraprocedural complications during lead extraction is well established. However, its utility as an active procedural tool in technically challenging cases remains less well defined. A growing body of literature supports the use of 3D and multiplane TEE monitoring in technically difficult transvenous lead extractions, describing improved intraprocedural visualization of lead position relative to intracardiac structures and enhanced procedural safety compared to fluoroscopy or 2D imaging alone [3,4]. This case illustrates the use of 3D-TEE to facilitate lead extraction in a high-risk patient.

## Case presentation

A 54-year-old male with nonischemic cardiomyopathy (ejection fraction <20% at this presentation) and end-stage renal disease on hemodialysis presented with methicillin-resistant *Staphylococcus aureus* (MRSA) bacteremia in the setting of a chronically implanted Abbott Quadra Assura MP™ CRT-D (Abbott Laboratories, Sylmar, CA, USA) biventricular implantable cardioverter-defibrillator (ICD), originally placed 13 years prior for advanced atrioventricular block and heart failure with reduced ejection fraction (HFrEF) (25%-30% at that time), with combined indications for permanent pacing and cardiac resynchronization therapy with defibrillator backup. Bacteremia was diagnosed on blood culture, which grew MRSA, in the setting of a chronic diabetic foot wound/cellulitis of the lower extremity as the presumed source. The patient's systemic presentation was relatively mild, without fever, chills, or hemodynamic instability, and blood cultures were obtained as part of routine evaluation of his known active foot infection rather than in response to overt signs of sepsis. Echocardiographic evaluation demonstrated severe systolic dysfunction and a mobile echodensity consistent with lead vegetation near the superior vena cava (SVC)-right atrial junction, necessitating complete device extraction. Given the patient's severe cardiomyopathy and active infection, complete extraction was attempted in a single operative session; however, one lead could not be fully removed despite mobilization efforts, necessitating a second procedure.

**Figure 1:**
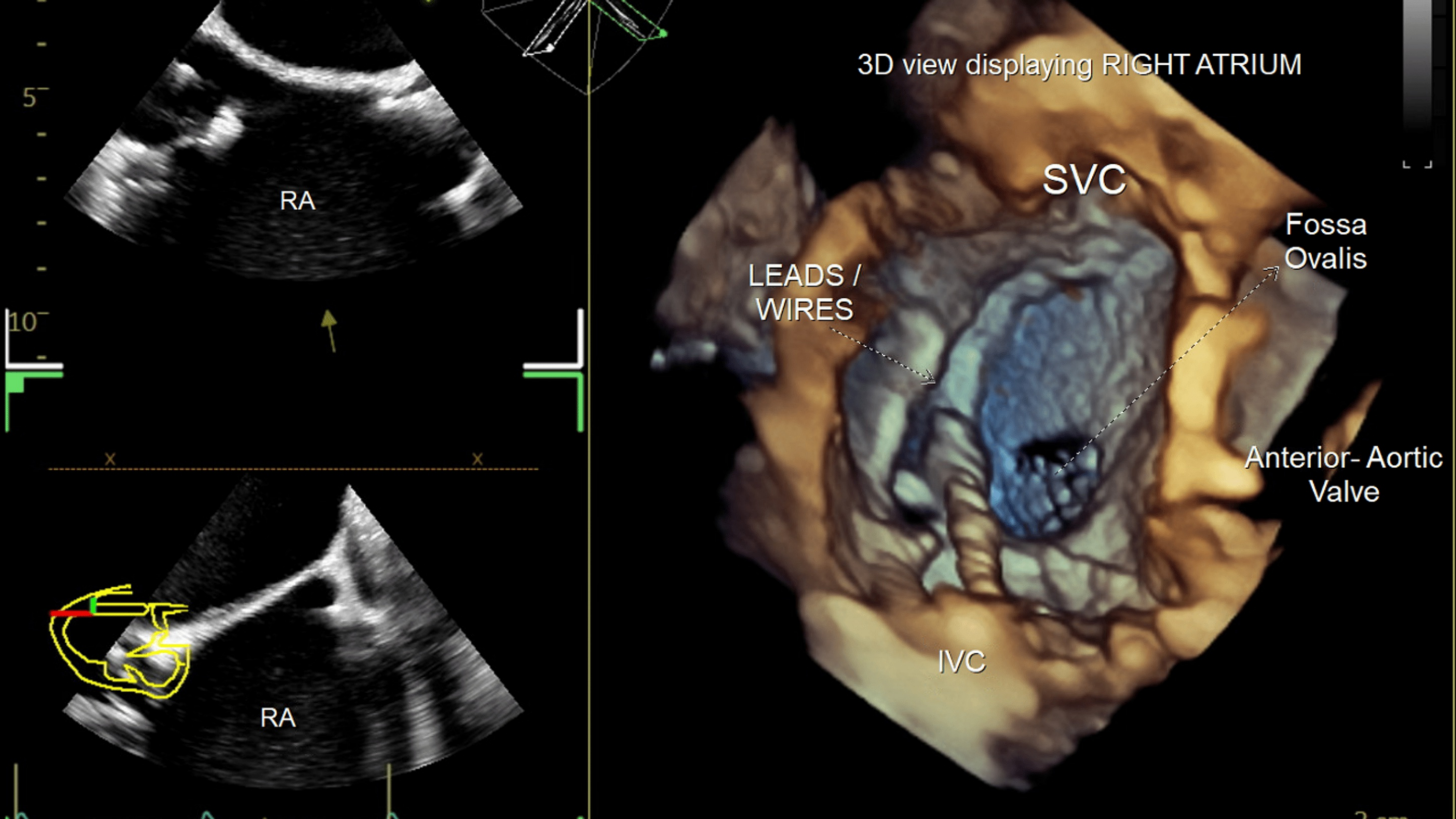
Three-Dimensional Transesophageal Echocardiographic Visualization of a Retained ICD Lead Within the Right Atrium Three-dimensional transesophageal echocardiography demonstrates the retained intracardiac lead traversing the right atrium with its relationship to adjacent structures, including the superior vena cava (SVC), inferior vena cava (IVC), and interatrial septum. Enhanced spatial visualization helped define lead orientation and intracardiac position before attempted snare retrieval. ICD: implantable cardioverter-defibrillator

**Figure 2:**
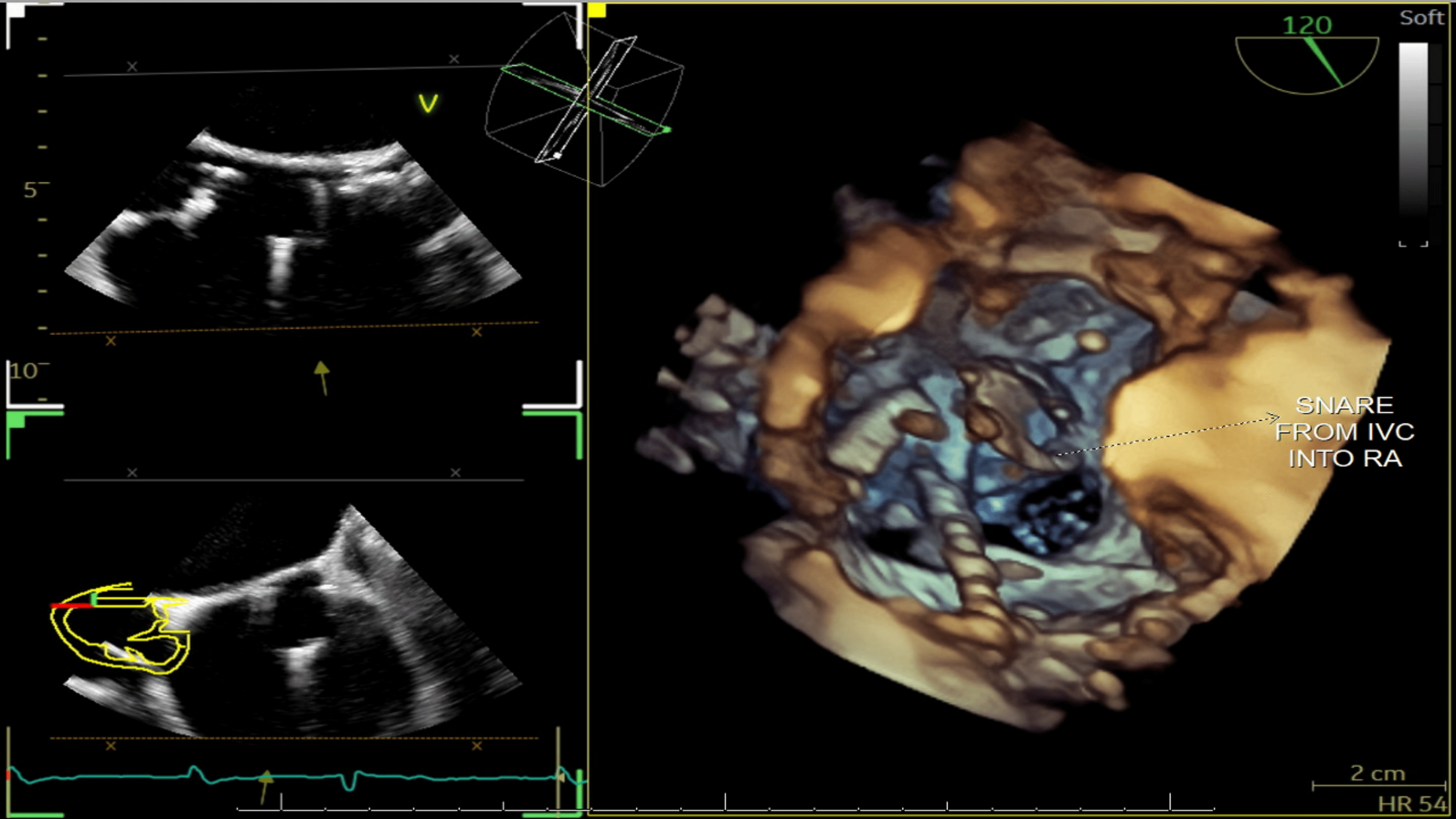
Three-Dimensional Transesophageal Echocardiographic Guidance of Femoral Snare Capture During Retained ICD Lead Extraction Three-dimensional transesophageal echocardiography demonstrates advancement of the femoral snare from the inferior vena cava into the right atrium during retrieval of the retained lead. Real-time spatial imaging facilitated precise snare positioning and successful capture of the lead after multiple conventional retrieval attempts were unsuccessful. ICD: implantable cardioverter-defibrillator

Initial laser-assisted transvenous lead extraction was performed in the operating room under general anesthesia with invasive hemodynamic monitoring and continuous TEE guidance, with a total fluoroscopy time of 21 minutes 33.8 seconds. Although portions of the lead were successfully mobilized, complete removal was not achieved, and a lead fragment was retained. The patient was maintained on targeted intravenous antimicrobial therapy for bacteremia throughout the interval between procedures. During this interval, the patient's ICD generator remained removed, and he was managed in the intensive care unit under continuous telemetry monitoring with external defibrillation capability immediately available. He required transient vasopressor support for hemodynamic instability, which was successfully weaned prior to the second procedure. Antimicrobial therapy was narrowed to vancomycin with renal dosing adjustments given his end-stage renal disease. Repeat blood cultures obtained six days after the initial procedure were negative, confirming clearance of bacteremia prior to the second extraction attempt. White blood cell count downtrended from 12.8 × 10³/µL on the day following the initial procedure to 5.7 × 10³/µL on the day of the second procedure, consistent with resolving inflammatory response. Baseline inflammatory markers obtained prior to the first procedure showed C-reactive protein 79.0 mg/L and erythrocyte sedimentation rate 84 mm/hr. Serum creatinine ranged from 2.63 to 3.82 mg/dL during the interval, and the patient continued routine hemodialysis throughout. Two weeks later, completion extraction was performed in the electrophysiology laboratory. Multiple conventional snaring attempts were unsuccessful due to the orientation and intracardiac adherence of the retained lead. Three-dimensional TEE was subsequently employed to obtain enhanced spatial visualization of the lead and surrounding intracardiac structures (Figure 1). Under 3D-TEE guidance, a femoral snare was advanced from the inferior vena cava (IVC) into the right atrium, successfully capturing and removing the retained lead without complication (Figure 2). Following discharge, the patient was followed by infectious disease for completion of intravenous antimicrobial therapy with weekly laboratory monitoring and by cardiology for outpatient management of heart failure, with device reimplantation deferred given ongoing infectious risk.

## Discussion

Transvenous lead extraction is the definitive treatment for CIED infection, but it carries procedural risks that are particularly pronounced in patients with severe cardiomyopathy and limited cardiopulmonary reserve [2]. Over time, implantable leads develop fibrotic adhesions to the venous and intracardiac structures they traverse. These adhesions significantly increase the risk of serious complications, including vascular injury, myocardial perforation, and cardiac tamponade. Patients with advanced heart failure are especially vulnerable, as even minor hemodynamic perturbations can precipitate clinical decompensation [5].

This case illustrates many of the challenges inherent to complex lead extractions. The patient harbored several compounding risk factors: severe nonischemic cardiomyopathy with a markedly reduced ejection fraction, end-stage renal disease on dialysis, active MRSA bacteremia, and a device implanted for over a decade. Prolonged lead dwell time is well recognized as a major determinant of extraction difficulty [6], as long-standing leads become fibrotically encapsulated and adherent to both vascular and intracardiac structures, simultaneously increasing technical complexity and the risk of hemodynamic instability. It is important to distinguish this scenario from delayed extraction in the sense described by prior literature [1], which refers to prolonged deferral of the decision to pursue extraction after CIED infection is diagnosed. In our patient, extraction was initiated promptly; however, continued forceful manipulation of the adherent lead during the first session was judged to carry a greater immediate risk of cardiac perforation or hemodynamic decompensation, given the patient's EF <20%, than a brief antibiotic-covered interval before a second attempt under improved conditions.

The initial procedure was performed in the operating room, where a controlled environment with full invasive monitoring, continuous TEE guidance, and immediate surgical backup was available [2]. Although one lead was successfully removed, the second lead could not be fully extracted despite mobilization efforts, and completion was deferred to a second procedure. This unplanned interval represented a high-risk window, given the retained infected lead fragment and the patient's severe cardiomyopathy and end-stage renal disease, rather than a recommended therapeutic strategy; this risk was mitigated, though not eliminated, by continuous intensive care unit monitoring and targeted antimicrobial therapy, with repeat blood cultures confirming clearance of bacteremia prior to the second procedure. Current guidelines classify retained infected hardware as a major procedural failure associated with substantially increased mortality compared to single-session complete extraction [2]; our case does not support staged extraction as a preferred strategy but illustrates that, when technical failure necessitates a delay, careful antimicrobial management and intensive monitoring can allow safe completion. The second-stage procedure was performed in the electrophysiology laboratory rather than a hybrid operating suite, as femoral snare retrieval is a fluoroscopically guided interventional technique rather than a surgical one; extensive invasive monitoring, vasopressor support, and TEE guidance were employed to mitigate risk, though immediate cardiothoracic surgical backup for emergency sternotomy was not formally in place. This represents an important consideration for future high-risk extractions of this nature. Complete procedural metrics, including fluoroscopy time and radiation dose for the second procedure, were not available for retrospective reporting and represent a limitation of this case.

Beyond the benefits of staging, advanced imaging was integral to the successful completion of the extraction. TEE provides real-time visualization of intracardiac structures during lead extraction, enabling early identification of complications such as pericardial effusion, ventricular dysfunction, and vascular injury. In this case, 3D-TEE provided superior spatial orientation of the retained lead within the right atrium compared to standard 2D imaging. Specifically, 3D-TEE allowed simultaneous visualization of the lead's course relative to fixed anatomic landmarks, including the SVC, IVC, fossa ovalis, and aortic valve, guiding precise snare trajectory from the IVC into the right atrium after fluoroscopically guided attempts alone had failed. Imaging was obtained from the mid-esophageal window using an en-face bicaval view (typically acquired at a multiplane angle of approximately 90°-110°), with 3D zoom acquisition of the right atrium to generate the volumetric reconstruction used for procedural guidance. Prior reports have demonstrated that 3D-TEE offers superior delineation of the spatial relationships between device leads and surrounding cardiac structures, facilitating more precise procedural guidance [3]. In retrospect, earlier use of 3D-TEE, rather than escalation only after failed conventional snaring attempts, may have reduced overall manipulation time and procedural risk in this high-risk patient and supports consideration of upfront 3D-TEE guidance in similarly complex extractions.

Leveraging this enhanced visualization, the procedural team was able to precisely direct a femoral snare, advanced from the IVC into the right atrium, to successfully capture and remove the retained lead. This case highlights the potential value of 3D-TEE as an adjunctive imaging modality in technically challenging lead extractions.

Taken together, these cases underscore the importance of multidisciplinary planning, staged procedural strategies, and advanced imaging in the management of complex CIED extractions. In high-risk patients with severe cardiomyopathy and long-standing leads, advanced echocardiographic guidance may enable successful extraction when conventional approaches have been exhausted.

## Conclusions

This case highlights a significant challenge associated with transvenous lead extraction in high-risk patients, particularly those with prolonged lead dwell times and unfavorable lead orientation. Three-dimensional TEE proved instrumental in providing the spatial orientation necessary to successfully remove the retained lead via femoral snaring when conventional techniques had failed, underscoring its potential to expand the procedural armamentarium in difficult extractions.

In this case, 3D-TEE served as an effective rescue modality after conventional fluoroscopy-guided snaring had failed. Based on this experience, we suggest that earlier consideration of 3D-TEE guidance, rather than reserving it until after failed conventional attempts, may be a reasonable strategy to explore in future high-risk cases with prolonged lead dwell times and severe cardiomyopathy, potentially reducing manipulation time and procedural risk.
